# Case Report: Central Retinal Artery Occlusion in a COVID-19 Patient

**DOI:** 10.3389/fphar.2020.588384

**Published:** 2020-12-23

**Authors:** Andrea Montesel, Claudio Bucolo, Victoria Mouvet, Emmanuelle Moret, Chiara M. Eandi

**Affiliations:** ^1^ Department of Ophthalmology, Fondation Asile des Aveugles, Jules Gonin Eye Hospital, University of Lausanne, Lausanne, Switzerland; ^2^ Department of Biomedical and Biotechnological Sciences, Section of Pharmacology, University of Catania, Catania, Italy

**Keywords:** severe acute respiratory syndrome coronavirus 2, coronavirus disease-2019, central retinal artery occlusion, thrombosis, case report, SARS-CoV-2

## Abstract

We report a case of central retinal artery occlusion (CRAO) in a patient with a previous history of severe COVID-19 disease. This disease has been associated with inflammatory-induced homeostasis changes leading to endothelial dysfunction and a procoagulant state with multi-organ involvement, but the burden of thromboembolic complications in COVID-19 patients is currently unknown. The pathogenesis of retinal artery occlusions is a multifactorial process where inflammation and hypercoagulation state are established risk factors. Even if our experience may represent a coincidental relationship, it is likely that COVID-19 patients could be at risk of developing retinal vascular occlusions. A focused ophthalmological surveillance is advisable to prevent and manage this possible cause of severe vision loss that has an important impact in health care system.

## Introduction

The ongoing pandemic of COVID-19 disease, caused by the severe acute respiratory syndrome coronavirus 2 (SARS-CoV-2), has been associated with inflammatory-induced homeostasis changes leading to a severe coagulopathy with multi-organ involvement (Levi et al., 2020). The pathogenesis of retinal artery occlusions (RAO) is a multifactorial process where inflammatory and hypercoagulation state are established risk factors (Flaxel et al., 2020). However, the effects of COVID-19 inflammatory and pro-coagulant state over the retinal vascular system have not been investigated yet. We report a case of a patient developing a central retinal artery occlusion after a severe SARS-CoV-2 infection.

## Cases Presentation

The patient was a 59-year-old male, African ethnicity, with a previous longstanding history of hypertension and hyperuricemia under treatment (Table 1). He was hospitalized four days after the onset of fever, dry cough and progressive dyspnea. On admission, nasopharyngeal samples tested positive for SARS-CoV-2 infection at polymerase chain reaction (PCR) molecular test and a diagnosis of COVID-19 disease was made. Blood test revealed elevated inflammatory markers and a procoagulant state (Table 2). The following day the conditions of the patient deteriorated and he was transferred to the intensive care unit (ICU). Here he was intubated to receive mechanical ventilation and he started a 5 days COVID-19 therapy with hydroxychloroquine 400 mg twice per day, lopinavir/ritonavir association (200 and 50 mg respectively) twice per day, and a single intravenous dose of tocilizumab 800 mg. The ICU stay was complicated by a bacterial pneumonia treated with broad-spectrum antibiotics and a stage 3 acute renal injury that required hemodialysis. The patient remained intubated for a total of 10 days, five of which with prone positioning, and he was later transferred to a neurorehabilitation department for physical deconditioning and mental confusion after the exclusion of cerebral ischemic events with computed tomography angiography (CTA) of the cerebral arteries and magnetic resonance imaging (MRI) of the brain.

**TABLE 1:** Patient’s clinical data on admission.

Age	59	Heart Rate	120 bpm
Sex	Male	Peripheral 0_2_ Saturation	77%
Height	174 cm	Blood Pressure	165/85 mmHg
Weight	99.4 kg	Temperature	39.1°C
Previous Medical History		Hypertension, Hyperuricemia	
Domiciliary Therapy		Amlodipine 10mg, Perindopril 10 mg, Torasemide 5mg, Allopurinol 100 mg	

**TABLE 2:** Coagulation and inflammatory markers on admission.

Marker	Level	References Range
Thrombin time	18 s	14–19 s
aPTT	43 s	26–37 s
Platelet count	109 g/L	150–350 g/L
D-Dimer	2.059 ng/ml	<500 ng/ml
Fibrinogen	5.9 g/L	2.0–4.0 g/L
White blood cell count	8.6 × 10^3^/µl	4–10 × 10^3^/µl
C-reactive protein	184 mg/L	<10 mg/L
IL-1 alpha	<1 pg/ml	<6 pg/ml
IL-1RA	14,725 pg/ml	<720 pg/ml
IL-6	42 pg/dl	<11 pg/dl
IL-7	7 pg/dl	<3 pg/dl
TNF-alpha	<12 pg/ml	<12 pg/ml

aPTT, activated partial thromboplastin time; IL, Interleukin; IL-1 RA; Interleukin-1 Receptor Antagonist; TNF, tumor necrosis factor.

**FIGURE 1:**
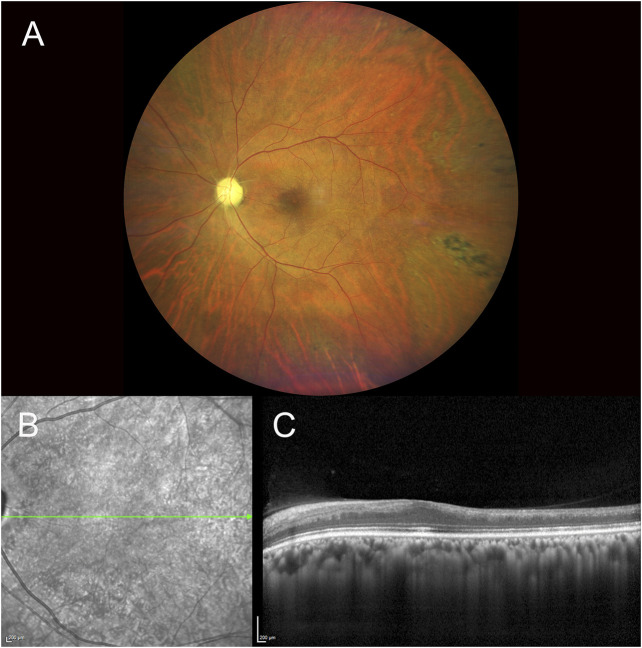
Fundus photography of the left eye showing the presence of a pale optic disc, diffuse arterial narrowing, a mild ‘cherry-red spot’ macula and peripheral areas of retinal pigmented epithelium hyperpigmentation **(A)**. IR and SD-OCT acquisition over the macular region of the same eye denoting atrophy of the inner retina layers with loss of foveal depression and temporal macular thinning **(B,C)** [IR, infrared reflectance, SD-OCT, *spectral domain optical coherence tomography*].

**FIGURE 2:**
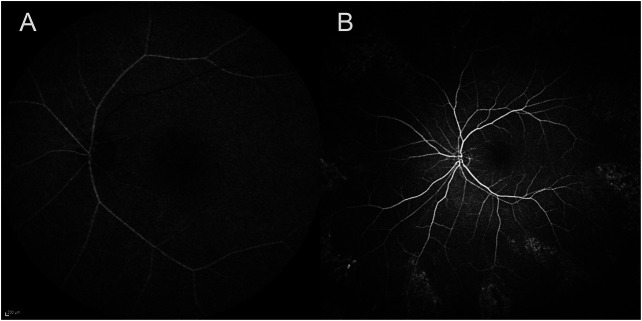
Fluorescein angiography of the left eye 5 days after the diagnosis of CRAO showing a delayed arrival of the dye in the eye 43 s after the injection **(A)**, peripheral areas of capillary nonperfusion, arteriovenous anastomoses, and neovascular ‘sea-fans’ 4 min and 22 s after the dye injection **(B)** [CRAO, central retinal artery occlusion].

**FIGURE 3:**
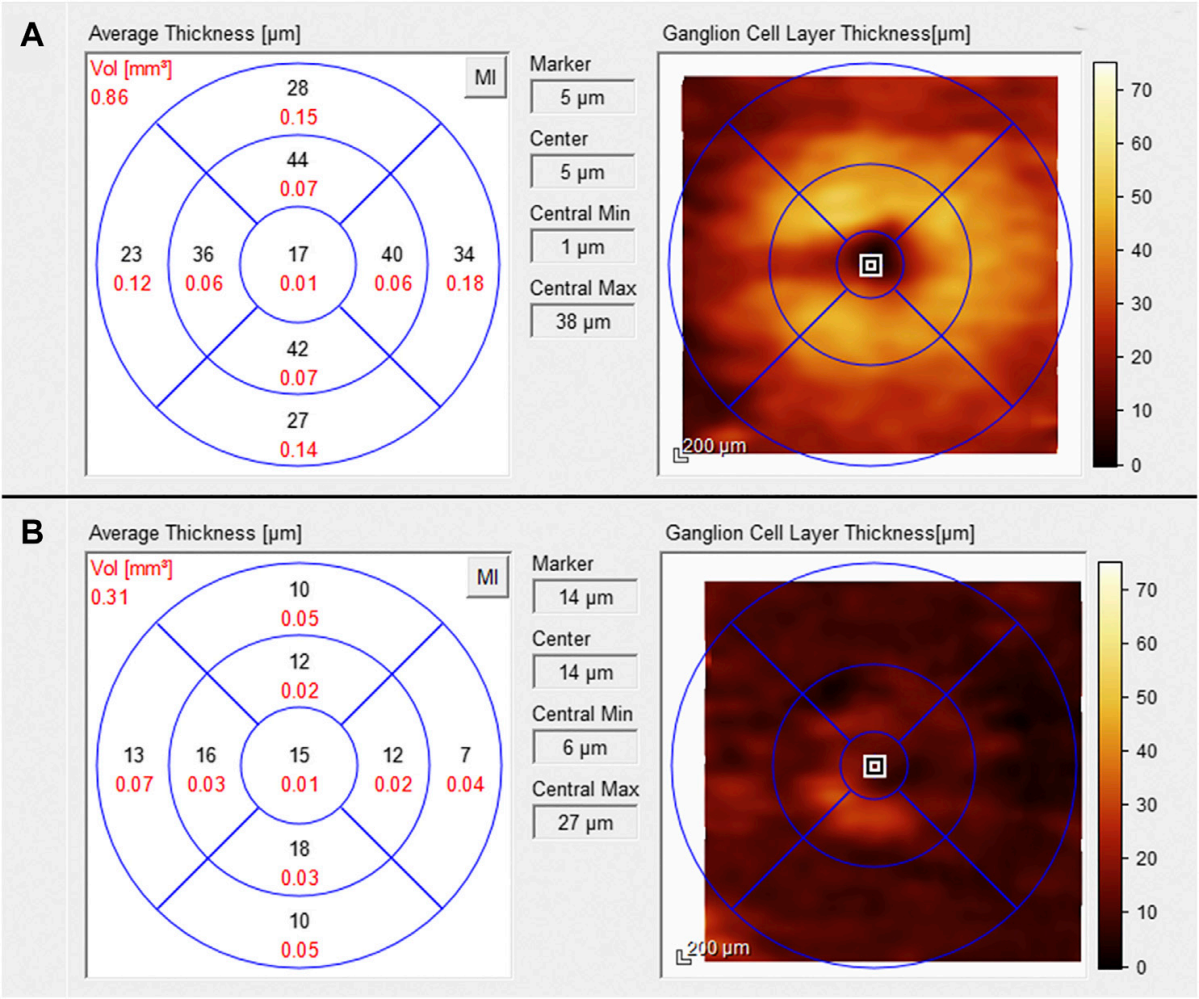
Ganglion cell layer thickness analysis performed with the SD-OCT one month after the arterial occlusion. Within normal limits in the right eye **(A)** and atrophic in the left eye **(B)** [SD-OCT, spectral domain optical coherence tomography].

One week after discharge from the hospital, he presented to our emergency service complaining of painless vision loss in the left eye. The patient noticed a decrease in visual function during his neurorehabilitation stay, but he did not realize how severe it was until he covered the right eye the day before. On examination, the best-corrected visual acuity (BCVA, Snellen equivalent) was 20/20 in the right eye and light perception in the left eye. Right eye ophthalmologic examination was unremarkable, while the left eye showed a nonreactive mydriasis, and dilated fundus ophthalmoscopy revealed the presence of severe arterial narrowing, retinal whitening in the macular region with loss of the physiological macular reflex and peripheral areas of retinal pigment epithelium hyperpigmentation (Figure 1A). Optical coherence tomography (OCT) showed temporal macular thinning in both eyes and severe atrophy of the inner retina layers with loss of the foveal depression in the left eye (Figures 1B,C). A diagnosis of CRAO was hence made. Fluorescein angiography (FA) performed 5 days later confirmed the diagnosis of CRAO revealing severe delay in the filling of the retinal arteries and a delayed arteriovenous transit time. In addition, findings consistent with sickle cell retinopathy were remarked, including areas of peripheral capillary nonperfusion, arteriovenous anastomoses, and neovascular “sea-fans” (Figure 2). Blood tests with hemoglobin electrophoresis confirmed the presence of heterozygous hemoglobin S (sickle cell trait), while the inflammatory markers (erythrocyte sedimentation rate and C-reactive protein) were within normal limits and not suggestive of an arteritic CRAO. The patient was further evaluated by the stroke unit with brain CTA and MRI of the encephalic trunk that did not reveal any acute sign of vascular events. At one-month follow-up visit, vision in the left eye improved to counting fingers and the left pupil was still nonreactive. OCT scans confirmed the loss of the foveal depression and showed ganglion cell layer atrophy (Figure 3).

## Discussion

The COVID-19 disease has been described to induce inflammatory-induced homeostasis changes that predispose to thrombotic disease in both venous and arterial circulation (Levi et al., 2020; Levolger et al., 2020). Post-mortem analysis showed evidence of direct viral infection of the endothelial cells and diffuse endothelial inflammation leading to endothelial dysfunction and a procoagulant state (Varga et al., 2020). The risk of thromboembolic events in COVID-19 patients is currently under investigation, with preliminary results showing significantly prolonged prothrombin time, high D-dimer levels and increased concentrations of proinflammatory cytokines and biomarkers of inflammation in patients with more severe disease, indicating the likelihood of disseminated intravascular coagulation or thrombotic microangiopathy (Connors and Levy, 2020; Levi et al., 2020). Even though most cases are affected by venous thromboembolism (Llitjos et al., 2020), there are increasing reports of COVID-19 induced arterial thromboembolic complications, highlighting the thrombogenicity of SARS-CoV-2 infection (Levolger et al., 2020). Lodigiani et al. (2020) in a cohort of 388 consecutive patients with laboratory-proven COVID-19 requiring hospital admission, disclosed a remarkable rate of venous and arterial thromboembolic complications of approximately 8% despite the use of anticoagulant prophylaxis (Lodigiani et al., 2020).

It is also well established that a considerable percentage of subjects infected by the coronavirus develop a mild infection or are asymptomatic, and in these cases the clinical and histopathology data available are limited. Hence, the burden of thromboembolic complications in COVID-19 patients is currently unknown, with some authors suggesting that it may represent an underestimated, large-scale issue (Lodigiani et al., 2020).

We report a case of a patient that developed a central retinal artery occlusion after a severe COVID-19 disease. CRAO are obstructions of retinal blood flow that result in severe vision loss. They are divided in arteritic CRAO, mainly due to giant cells arteritis, and non-arteritic CRAO, where platelet fibrin thrombi or emboli as a result of atherosclerotic disease are responsible for over two-third of all CRAO cases (Varma et al., 2013). However, inflammatory and pro-coagulant state could also lead to the development of retinal vascular occlusions (Flaxel et al., 2020). Therefore, we might hypothesize that in the case we reported the induction of a prothrombotic vascular endothelial microenvironment by the COVID-19 disease could have represented a precipitating factor causing the development of a CRAO, similar to what observed in other organs such as myocardial and renal microvasculature of COVID-19 patients (Gheblawi et al., 2020).

Retinal vascular damage could be correlated to the ocular expression of the angiotensin-converting enzyme 2 (ACE2). SARS-CoV-2 is internalized by human cells after binding with the ACE2, which acts as a functional receptor for the virus. The expression of ACE2 has been detected in human aqueous humor and in retinal tissue, particularly in pigmented epithelial cells, photoreceptors and Müller cells. ACE2 plays an important role in the vasoprotective axis of the renin–angiotensin system (RAS) as it counterbalances the vasoconstrictive, proliferative, fibrotic and proinflammatory effects of the ACE–Ang II–angiotensin II type 1 receptor (AT1R) axis generating Angiotensin 1–7 (Ang 1–7).

COVID-19 patients show a depletion of ACE2 function directly following the binding with SARS-CoV-2 and indirectly *via* shedding and proteolytic processing (Gheblawi et al., 2020). This loss of ACE2 has several known deleterious effects. In fact, there is an increase of Ang II and a decrease of Ang 1–7 levels in tissues leading to exacerbation of hypertension. Moreover, genetic ACE2 deficit correlates to an upregulation of proinflammatory stimuli and atherogenesis mediators in animal models, suggesting a key role of ACE2 in blocking vascular inflammation and atherosclerosis (Gheblawi et al., 2020). Furthermore, in animal models of diabetic retinopathy, ACE2 overexpression inhibits the up-regulation of proinflammatory factors and adhesion molecules in the retinal circulation, while ACE2 deficiency is associated with profibrotic and proinflammatory microvascular dysfunction and retinal nerve fiber layer infarcts (Dominguez 2nd et al., 2016; Duan et al., 2018).

**FIGURE 4:**
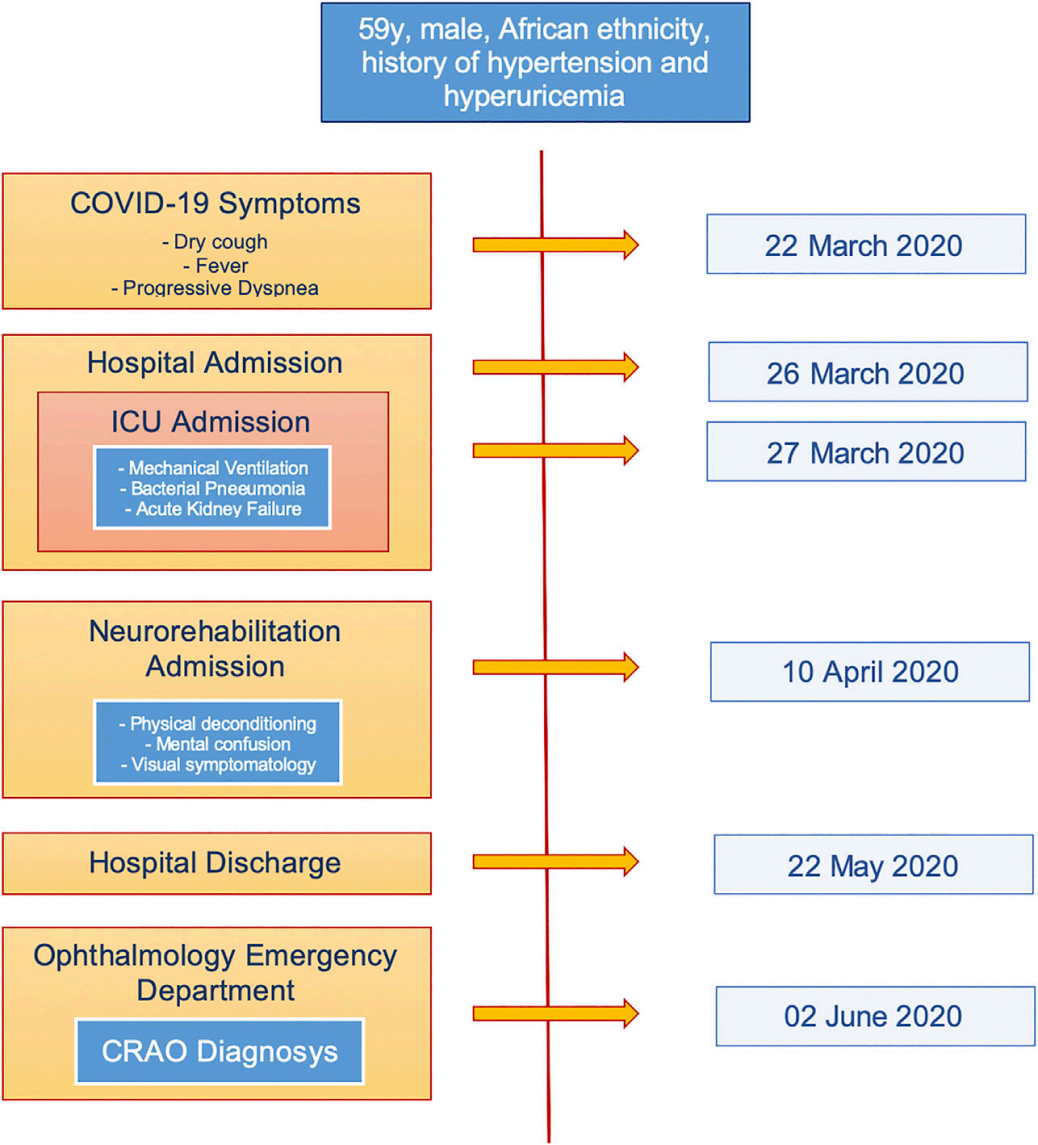
Case report timeline.

To our knowledge, there are only two reports (Acharya et al., 2020; Dumitrascu et al., 2020) of patients who developed a retinal arterial occlusion in association with COVID-19 disease. In the first report, the CRAO developed during the second week of patient’s hospitalization, 3 days after the discharging from the ICU, where the patient received COVID-19-directed therapy including hydroxychloroquine, azithromycin, and tocilizumab. In the second case, the onset of the CRAO was in the sixth week of hospitalization and the patient was under anticoagulant therapy with apixaban after being treated with hydroxychloroquine, tocilizumab and enoxaparin. In both cases, the authors did not record the clinical findings with any imaging modality but reported only the fundus examinations findings. In our case, the exact timing of the CRAO could not be established, but we can assume that it developed during the hospitalization when the patient first reported the visual symptomology (Figure 4). In all three cases the patients had received treatment with hydroxychloroquine and tocilizumab prior to the CRAO onset. We performed a research on the PubMed database and we did not find any association between these two drugs and the induction of thrombosis. Chronic use of hydroxychloroquine could induce retinal toxicity, but its short-term usage on COVID-19 patients is unlikely to induce retinal damage (Marmor, 2020). Moreover, hydroxychloroquine has shown anti-thrombotic effects on patients affected by antiphospholipid syndrome (Kravvariti et al., 2020).

Even though it is not possible to assert with absolute certainty the association between the CRAO and COVID-19 disease, we have to highlight that the correlation is reasonable. Incidentally, the patient was diagnosed with sickle cell disease, and this condition could elicit retinal vascular occlusions, however these occlusions occur predominantly in small caliber peripheral arterioles and its association with CRAO is rare (Fine et al., 2000). We might speculate that the inflammatory and procoagulant state secondary to the COVID-19 could be the trigger for the retinal artery occlusion in patients with cardiovascular risk factors.

In conclusion, because the association between thrombosis and COVID-19 is becoming clearer, there are unresolved questions regarding its possible impact on retinal pathophysiology, especially in a population with multiple risk factors that might develop an asymptomatic SARS-CoV-2 infection, and for which the routine ophthalmic monitoring has been suspended (Recommendations for Urgent and Nonurgent Patient Care, 2020). However, the effects of COVID-19 inflammatory and pro-coagulant state over the retinal vascular system are currently unknown. A focused post-pandemic ophthalmological surveillance is advisable to prevent and eventually manage this possible cause of retinal damage and severe vision loss avoiding a negative impact in health care system.

## Data Availability Statement

The raw data supporting the conclusions of this article will be made available by the authors, without undue reservation.

## Ethics Statement

Written informed consent was obtained from the individuals for the publication of any potentially identifiable images or data included in this article.

## Author Contributions

All authors listed have made a substantial, direct, and intellectual contribution to the work and approved it for publication.

## Conflict of Interest

The authors declare that the research was conducted in the absence of any commercial or financial relationships that could be construed as a potential conflict of interest.
